# Draft genome sequences of two *Micromonospora* strains isolated from the root nodules of *Alnus glutinosa*

**DOI:** 10.1128/mra.01131-23

**Published:** 2024-02-01

**Authors:** Ryan Michael Thompson, Edward M. Fox, Maria del Carmen Montero-Calasanz

**Affiliations:** 1 School of Natural and Environmental Sciences, Newcastle University, Newcastle upon Tyne, United Kingdom; 2 Department of Applied Sciences, Northumbria University, Newcastle upon Tyne, United Kingdom; 3 IFAPA Las Torres-Andalusian Institute of Agricultural and Fisheries Research and Training, Junta de Andalucía, Cra. Sevilla-Cazalla, Seville, Spain; The University of Arizona, Tucson, Arizona, USA

**Keywords:** *Micromonospora*, endophytes, *Alnus glutinosa*, *Alnus*, genomes

## Abstract

In this paper, the draft genomes of *Micromonospora* RTGN7 and RTP1Z1, derived from *Alnus glutinosa* root nodules, are reported. The assembly of RTGN7 is 6.6 Mbp, composed of 59 contigs, with an N_50_ of 321,872. RTP1Z1’s assembly is 6.3 Mbp, composed of 151 contigs, with an N_50_ of 76,442 bp.

## ANNOUNCEMENT

*Alnus glutinosa* nodules are hosts of *Micromonospora*, with isolation of two strains reported (1, 2). Isolation was conducted from the nodules of a single *A. glutinosa* within Saltwell Park, United Kingdom (54.944723–1.605852). Nodules were washed with tap water, and 4–15 lobes were surface sterilized in 1 mL of 25% strength household bleach (~1.125% sodium hypochlorite) for 5 minutes. After removing the bleach, the lobes were washed five times in 1 mL of sterile distilled water and homogenized in 0.5 mL of ¼ strength Ringer’s solution. To the homogenate, 14.5 mL of ¼ strength Ringer’s solution was added and plated upon tap water yeast extract agar (https://www.dsmz.de/microorganisms/medium/pdf/DSMZ_Medium1625.pdf) and Zhang starch soil extract agar (both containing 50 mg/mL nystatin) and incubated at 28°C (3).

RTGN7 and RTP1Z1 were recovered from tap water yeast extract and Zhang starch soil extract, respectively, after 30 and 40 days of respective incubation. Single colonies were streaked upon their corresponding media and incubated at 28°C for 24 and 14 days individually, with three subculturing iterations performed from single colonies.

From a 10-day old culture of RTGN7, grown upon GYM agar (https://www.dsmz.de/microorganisms/medium/pdf/DSMZ_Medium65.pdf), DNA was extracted using a GenEluteBacterial Genomic DNA Kit (Sigma-Aldrich, USA) with an additional ethanol precipitation step. Sequencing was conducted by Novogene Co. Ltd., with a DNA library prepared using a Novogene NGS DNA library prep set (catalog no. Pt004), in which the DNA was randomly sheared into short fragments, end repaired, A tailed then ligated with the Illumina adaptor. These sequences were amplified using PCR, size selected for 350 bp, purified, and then sequenced using 150-bp Illumina paired-end sequencing upon an Illumina NovaSeq, with raw reads filtered using FastP (version 0.23.1) (4).

From an 11-day-old nutrient broth culture of RTP1Z1, DNA extraction and sequencing were conducted by MicrobesNG (http://www.microbesng.com). The material was incubated in 120 µL of TE buffer containing lysozyme (0.1 mg/mL) and RNase A (0.1 mg/mL) at 37°C for 25 minutes. After which, proteinase K and SDS (final concentration 0.1 mg/mL and 0.5%, respectively) were added and incubated for 5 minutes at 65°C. Genomic DNA was purified using equal volume solid-phase reversible immobilisation beads and elution buffer, with a DNA library prepared using a Nextera XT library prep kit (Illumina, USA). Two modifications were made according to the manufacturer’s protocol, the DNA amount was increased twofold, and the PCR elongation was extended to 45 seconds. The library was sequenced using a 250-bp paired-end protocol upon an Illumina NovaSeq 6000. The reads were trimmed using Trimmomatic (version 0.30) with a sliding window quality cutoff of Q_15_ (5).

The following analysis was conducted using the program’s standard settings unless otherwise noted. Both isolates reads were uploaded to Galaxy Europe (https://usegalaxy.eu/) (6), assessed using FASTQC (Galaxy Version 0.73 + galaxy0) (http://www.bioinformatics.babraham.ac.uk/projects/fastqc/), and assembled using Shovill (Galaxy Version 1.1.0 + galaxy1) (https://github.com/tseemann/shovill), and the assembly quality was assessed using Quast (Galaxy Version 5.0.2 + galaxy5) (7–9), with contigs <200 bp removed. The taxonomic relationship of RTGN7 and RTP1Z1 to their nearest neighbours was determined using TYGS (v389) (10, 11) (Table 1).

**TABLE 1:** Summary of read and assembly statistics of the isolates RTGN7 and RTP1Z1

Isolate	Raw read number	Filtered read	Assembly length (bp)	Contig number	N_50_ value	GC%	Estimated sequencing depth	Nearest neighbor (percentage identity)
RTGN7	9,080,972	9,041,320	6,649,740	84	321,872	72.18	205×	*Micromonospora rubida* NEAU-HG-1 (37.8%)
RTP1Z1	1,332,626	1,253,492	6,386,282	151	76,442	72.28	66×	*Micromonospora kangleipakensis* DSM 45612 (48.2%)

## Data Availability

The draft genomes of *Micromonospora* sp. RTGN7 and RTP1Z1 were deposited in INSDC under the accession numbers JAPZLF000000000 and JAPFQV000000000, respectively. The SRA data of *Micromonospora* sp. RTGN7 and RTP1Z1 were deposited in INSDC under the accession numbers SRR22064616 and SRR22354992, respectively.
